# Automated Detection of the Arterial Inner Walls of the Common Carotid Artery Based on Dynamic B-Mode Signals

**DOI:** 10.3390/s101210601

**Published:** 2010-11-29

**Authors:** Da-Chuan Cheng, Arno Schmidt-Trucksäss, Chung-Hsiang Liu, Shing-Hong Liu

**Affiliations:** 1 Department of Biomedical Imaging and Radiological Science, China Medical University, Taichung, Taiwan; 2 Institute of Exercise and Health Sciences, University of Basel, Basel, Switzerland; E-Mail: arno.schmidt-trucksaess@unibas.ch; 3 Department of Neurology, China Medical University Hospital, Taichung, Taiwan; E-Mail: greengen@gmail.com; 4 Department of Computer Science and Information Engineering, Chaoyang University of Technology, Taichung, Taiwan; E-Mail: shliu@cyut.edu.tw

**Keywords:** intima, media, adventitia, CCA, plaque

## Abstract

In this paper we propose a novel scheme able to automatically detect the intima and adventitia of both near and far walls of the common carotid artery in dynamic B-mode RF (radiofrequency) image sequences, with and without plaques. Via this automated system the lumen diameter changes along the heart cycle can be detected. Three image sequences have been tested and all results are compared to manual tracings made by two professional experts. The average errors for near and far wall detection are 0.058 mm and 0.067 mm, respectively. This system is able to analyze arterial plaques dynamically which is impossible to do manually due to the tremendous human workload involved.

## 1. Introduction

Carotid atherosclerosis may appear as intima-media thickening or plaque formation. Common carotid artery intima-media thickness (CCA-IMT) measurements have been confirmed to be an early marker of atherosclerosis [1] and have been associated with a higher risk of stroke [2] and myocardial infarction [3]. The non-invasive sonographic examination has demonstrated its potential in early prediction of cardiovascular diseases [4]. Carotid plaque formation is a different form of atherosclerotic damage of the carotid artery. An increasing grade of stenosis caused by plaques is associated with higher incidence of stroke and major cardiovascular events. Further, total plaque area is a major determinant of cardiovascular events and mortality.

B-mode sonography is widely used to scan the carotid artery in the diagnosis of atherosclerosis. It is noninvasive and low cost. The carotid IMT can be measured either manually [5], semi-automatically or automatically [6–14]. Carotid plaques are usually measured manually by measurement of the largest diameter in length and height, respectively. This can only be seen as a rough estimation of the plaque area. Measurement of stenosis degree is mostly performed via few points placed manually at the nearest point of the stenosis.

The automatic methods of outlining the outer boundaries of IMT and plaques have the potential to make results reproducible and eliminate the strong variations observed in manual tracings by different observers. Particularly, it is almost impractical to trace the boundaries of the inner wall on image sequences manually since this requires a tremendous amount of time. By using an automatic system the processing time can be considerably reduced. The motivation of this study was to develop a fiable system which is able to automatically detect the intima and adventitia of both near and far artery walls along a section, with or without plaques, using dynamic B-mode sonographic image sequences even under strong speckle noise conditions. This system can identify not only the IMT but also the lumen diameter (**LD**) during systolic and diastolic cycles, from which the artery’s volume change with respect to time can be outlined. A precise measurement of the IMT over the heart cycle in areas without plaque formation is the basis for the measurement of the local arterial stiffness. The same measurement in the area of a plaque offers the possibility of measuring the deformation of a plaque over the heart cycle, giving the perspective to analyze the deformability of a plaque. This may be a step forward of the detection of vulnerable plaques. Via this system, the dynamic processes of the carotid artery (**CA**) can be represented by some parameters such as IMT variation, LD variation, and the frequency analysis on the LD changes.

The carotid artery stiffness (or elasticity, **CS**) is one of the important factors for predicting cardio-vascular events [15,16]. In order to measure CS one has to calculate the cross-sectional area changes of the CCA during the systolic and diastolic cycle, *i.e.*, CDist = ΔA/(A·ΔP), where A is diastolic lumen area and ΔA is the maximum area change during a systolic-diastolic cycle, and ΔP is the local blood pressure change measured by a sphygmometer on branchial artery. The CDist can be converted to CS by giving CS = (CDist)^−½^ [15]. Our method is an automated scheme which is able to calculate the diameter changes of CCA during the heart beat cycle. It provides direct information for calculating ΔA and A.

In previous studies CS was calculated by measuring the LDs in systolic and diastolic states, *i.e.*, only two points. The contribution of this study is to propose a fiable technique for detecting the sectional IMT and LD changes along a section of CCA with respect to time, with and without plaques and even having speckle noises. This method is in general different from previously published works and it provides good results. Technically, we use a novel dual dynamic programming combined with some anatomic knowledge incorporated into the scheme which makes the detection more robust towards the presence of speckle noises.

## 2. Materials

After at least 15 minutes of rest in the supine position, ultrasonic examinations of the right and left CCA were performed. An Esaote Mylab 25 (Florence, Italy) ultrasound scanner with a transducer (LA523) having variable-band linear array (13–14 MHz). The neck of the study subjects was turned slightly to the left or right side for scanning the right or left carotid artery, respectively. The transducer was positioned at the anterior-lateral side of the neck. The lumen was maximized in the longitudinal plane with an optimal image of the near and the far vessel wall of the CCA or carotid bifurcation. Thus, typical double lines could be seen as the intima-media layer of the artery. Plaques were scanned in a longitudinal and cross-sectional plane showing the highest diameter. At least three heart cycles of every subject were acquired for measurement of the IMT or plaque, respectively. All sequences were stored digitally in the ultrasound device and transferred to a personal computer for further image analysis. This study complies with the Declaration of Helsinki and was approved by the ethics committee of the Technical University Munich, Germany. All patients signed an informed consent before being examined.

## 3. Methods

### 3.1. Problems Statement

The problems encountered in this study can be itemized as follows:

- Speckle noises;
- Calcification deposition on intima;
- Moving target;
- Parameter determination in different sonographic modalities.

The first problem is very common in the near arterial wall. Due to the jump of echo intensity between intimal surface and lumen, echoes (speckle noises) are transferred to the lumen which cause blurring of the intima-lumen interface. The second problem is that calcification deposition generally causes a shadow area under it. This results in a problem in detecting the far wall adventitia or the near wall intima depending on the location of the plaque. The third problem states that the artery changes in lumen diameter, IMT and position with systolic and diastolic phases. Since sequential images are processed, the tracking problem appears. The last problem is very common in many algorithms. Due to different properties of different ultrasonic machines, one has to determine the optimal parameter set for each specific machine.

### 3.2. Image Feature

**Figure 1.**
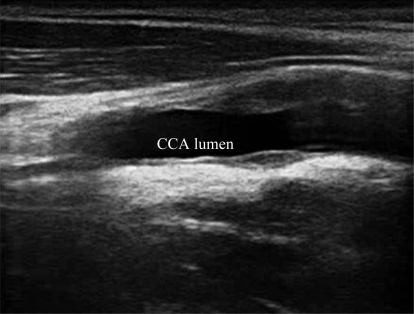
A sub-image from a B-mode sonographic image sequence. There are plaques on both near and far walls. (Sequence S1: Male, 36 yr old).

**Figure 2.**
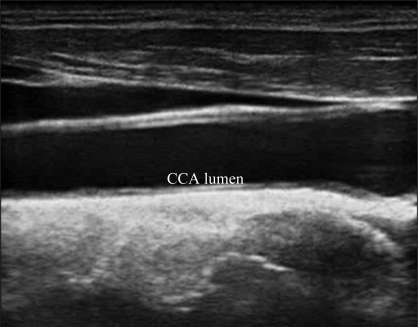
A sub-image from a B-mode sonographic image sequence. This patient has thin IMT. (Sequence S2: Male, 56 yr old).

**Figure 3.**
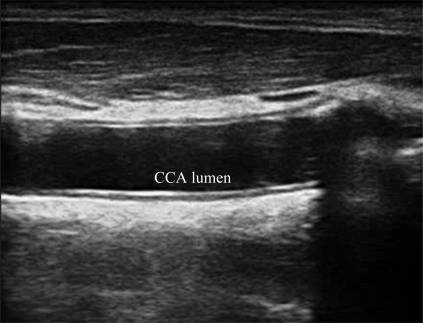
A sub-image from a B-mode sonographic image sequence. This patient has thick IMT. (Sequence S3: Male, 59 yr old).

A typical B-mode image is shown in Figure 1. There are plaques on both near and far walls. On the contrary, a typical CCA image without plaque having thin and thick IMT is shown in Figures 2 and 3, respectively. Outlining the boundaries of intima and adventitia is not easy, especially on the near wall. Some features have been studied in previous studies [7,11], in which the most frequently used feature is the gradient of gray-values. From our previous studies [7,14,17], it is found that MacLeod operator combined with some specific enhancement filters are very suitable for feature extraction in intima and adventitia detections.

The dynamic B-mode sonographic sequences are recorded and converted to a series of static images and saved in BMP format. A graphic user interface (GUI) is designed for the user to select a region of interest (ROI) for the following automatic analysis. The selected ROI is a rectangular area which is large enough to cover the possible area during a complete systolic and diastolic cycle. Similarly, when the IMT is analyzed, the rectangular area covers only the intima, adventitia, and part of artery lumen area. However, since the IMT does not change too much during the heart cycle, the rectangular area for IMT measurement is much smaller than the one for lumen diameter measurement. After the ROI is selected, the following analysis and measurement processes are fully automatic.

The feature image (*F*) is obtained from the ROI sub-image by means of the following filtration:

(1) $$F=|I*f_{M}|+I*f_{x}$$

where *I* denotes the ROI sub-image; *f_M_* is the MacLeod operator; and *f_x_* is the enhancement filter. The design of *f_x_* for the IMT and LD detection is different. The MacLeod filter is defined as follows [18]:

(2) $$f_{M}(x,\;y)=\text{exp}(-\frac{x^{2}+y^{2}}{d_{c}^{2}})[\text{exp}(-{\left(\frac{d_{xy}+d_{pk}}{d_{pk}}\right)}^{2})-\text{exp}(-{\left(\frac{d_{xy}-d_{pk}}{d_{pk}}\right)}^{2})]$$

where:

*d_c_* constant; determining the rate of radial decay,
*d_pk_* distance from axis to peaks in positive and negative weights,
*d_xy_* = *x* sin *θ* – *y* cos *θ*, perpendicular, distance of point (*x,y*) from axis of edge (at orientation *θ* to horizontal).

The MacLeod filter is used to detect the gradient in different orientations. In total we used four angles, *i.e.*, *θ* = 0, *π/*4, *π/*2, and 3*π/*4. However, due to the noise interference in the sub-intima area, a single MacLeod filter cannot finish this work. Therefore, we add another enhancement filter to reduce the effect of noises. This is because the gradient direction is considered in the enhancement filter to degenerate the noises effect in the sub-intima computed by MacLeod filter. There are two kinds of *f_x_*, one for IMT detection and another one for LD detection. Each one has its own duality, one for near wall and another one for far wall. The enhancement filter for far wall LD detection has been published in [17]. For simplicity we do not repeat it again because it is a large matrix. The enhancement filter for far and near wall IMT detection is as follows:

(3) $$f_{\text{IMT}(\text{far})}=\left[\begin{array}{ccccccc} 0 & 0 & 0 & -1 & 0 & 0 & 0\\ 0 & 0 & 0 & -1 & 0 & 0 & 0\\ 0 & 0 & 0 & -1 & 0 & 0 & 0\\ 0 & 0 & 0 & 0 & 0 & 0 & 0\\ 0 & 0 & 0 & 1 & 0 & 0 & 0\\ 0 & 0 & 0 & 1 & 0 & 0 & 0\\ 0 & 0 & 0 & 1 & 0 & 0 & 0 \end{array}\right],\;f_{\text{IMT}(\text{near})}=\left[\begin{array}{ccccccc} 0 & 0 & 0 & 1 & 0 & 0 & 0\\ 0 & 0 & 0 & 1 & 0 & 0 & 0\\ 0 & 0 & 0 & 1 & 0 & 0 & 0\\ 0 & 0 & 0 & 0 & 0 & 0 & 0\\ 0 & 0 & 0 & -1 & 0 & 0 & 0\\ 0 & 0 & 0 & -1 & 0 & 0 & 0\\ 0 & 0 & 0 & -1 & 0 & 0 & 0 \end{array}\right]$$

**Figure 4.**
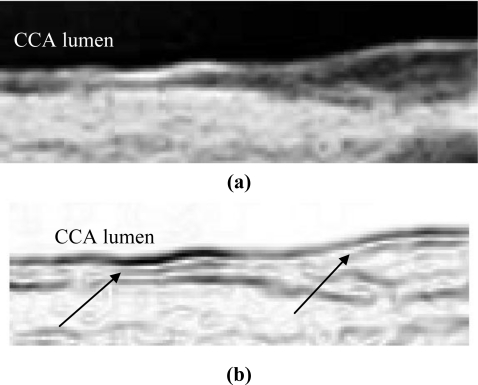

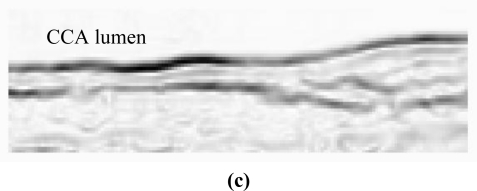
**(a)** Original sub-image. **(b)** Result made by MacLeod filter. There is a noise layer in the sub-intima indicated by arrows. **(c)** Result made by the combination of MacLeod and enhancement filters.

Figure 4 demonstrates the noise reduction effect of the enhancement filter. In cases without plaques, the thickness of sub-intima does not change on a large scale. If it has plaques, the noise layer existing in the sub-intima is not so strong and therefore, it can be ignored. The size of the filter is based on the consideration of the sensor’s frequency and the anatomic structure. Since the normal IMT is around 0.5 mm for young persons and the sensor is 10 MHz, therefore, the pixel size is about 0.1 mm and the IMT is around 5 pixels. That is the reason why the filter size is 7 × 7. Via this design, the IMT can be detected with less interference from the noise layer.

On the task of lumen diameter detection, detecting the intima of both near and far walls precisely is very important. To achieve this goal, we separate the detection into two phases, *i.e.*, the near wall IA (intima-adventitia) and far wall IA detection. From the two detections the intima layers of both walls can be obtained. The reason for using DDP (Dual Dynamic Programming) two-times instead of using the 4-dynamic programming is because it requires less computation time.

Most previous studies use the adventitia of the near wall instead of the initima to calculate LD. This is an approximation when there is no plaque. Since some patients have plaques, this approximation might cause significant faults in LD calculation.

**Figure 5.**
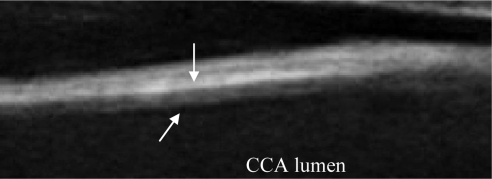
A sub-image in the near wall. There are some noises under the intima resulting difficulties in detection. The oblique arrow indicates the intima and the vertical arrow indicates the outer wall of the CA. (Sequence S2).

According to our experience the enhancement filter in (2) combined with MacLeod filter produces good results for the far wall IMT detection. However, it is not the case for the near wall. Figure 5 demonstrates one typical problem. This image is a sub-image extracted from a raw image in a sequence. Some noises existed under the intima when the artery is in the systolic cycle because its larger moving speed. This will cause ambiguity in IA detection because most of the rest images have no such kind of noises. We therefore propose a method to alleviate this problem.

The ambiguity results from the fact that the directional gradient of gray values cannot differentiate the interface between the lumen-intima interface and lumen-noise interface. If the gradient of the interface between the noise and artery lumen is larger than that between media and adventitia (Figure 5 shows the case), then the following DDP will have a faulty result. The problem then becomes how to enhance the interface between media and adventitia. We observed that there are large gray values under both lumen-intima and media-adventitia interfaces in the near wall. We can make good use of this information. Nevertheless, we do not hope to enhance the outer wall simultaneously, since it will result in ambiguity in searching the dual lines, *i.e.*, the intima and the adventitia. To solve this problem we apply dynamic programming to find out the outer wall first. Then an additional feature image (H) is calculated as follows:

(4) $$H(y,\;x)=1-\frac{1}{r}\sum_{i=y+1}^{y+r}I(i,\;x),\;\;\;\text{for}\;\;M-r>y>y_{\text{outer}},\;\;N\geq x\geq 1$$

where *I* is the sub-image of the size M × N and *r* is determined by the knowledge and the image resolution; *y_outer_* is the coordinate of the outer wall. Notably, *I* is normalized so that 0 ≤ *I*(*y,x*) ≤ 1.

### 3.3. Dual Dynamic Programming (DDP)

In our previous study we have developed dual dynamic programming (DDP) for IMT detection. Here we briefly describe this scheme as follows. Let H(x, y) denote the feature image grid of size M × N, where M and N are the number of rows and columns, respectively. Assume the DDP running from left to right in order to find dual curves such as intima and adventitia (or intimal layers of both near and far walls in case of detecting the lumen diameter). The DDP intends to find the global maximum, which is the summation of the feature values on the grid where the dual curves go through. The dual curves can be denoted as a point set {(*x, y*_1_), (*x, y*_2_)}, 1 ≤ *x* ≤ *N*, and its corresponding feature values are {*H*(*x, y*_1_), *H*(*x, y*_2_)}. The anatomic knowledge is then embedded into the DDP structure:

- Quasi-parallel property: The intima and adventitia do not cross each other. Only in very limited cases such as when the intima is damaged so that adventitia might be exposed to the artery lumen. Based on this fact, the relationship is defined to be 1≤*y*_1_≤*y*_2_≤*M*.
- Minimum- and Maximum-Thickness property: The IMT has normally 0.5 mm. However, it varies and depends on age and diseases. Elderly people or CVD (cardiovascular disease) patients have larger IMT than younger or normal persons. Plaques are often a symptom seen in artery walls. Based on this finding, the minimal and maximal thickness can be given as *d*_min_ and *d*_max_, respectively. They have to satisfy a constraint: *d*_min_ ≤ *y*_2_ – *y*_1_ ≤ *d*_max_. These two parameters are determined by the image pixel size and related to the selected ROI size. In which *d*_min_ is normally set to 0.4 mm and *d*_max_ can be set smaller if there is no plaque in order to speed-up the IMT detection process. In the LD detection, *d*_max_ has to be set larger since the LD is larger than IMT.
- Smoothness property: The last consideration is smoothness. It is assumed that intima or adventitia does not change abruptly. There are two terms defined in the energy function, *i.e.*, the single line smoothness (*d_r_* =|*y_x_* – *y_x_*_–1_|) and dual-line change smoothness (Δω*_x_* =|ω*_x_* – ω*_x_*_–1_|), where ω*_x_* =|*y*_2,_*_x_* – *y*_1,_*_x_*|.

Finally, the cost function can be defined to be:

(5) $$J(x,\;y_{1},\;y_{2})=\underset{\begin{array}{l} j_{1},j_{2}\in \\ \left\{-k,\ldots ,k\right\} \end{array}}{\text{min}}\{J(x-1,\;y_{1}+j_{1},\;y_{2}+j_{2})+{\alpha \Delta \omega }_{x}+\beta (|j_{1}|+|j_{2}|)\}+H(x,\;y_{1})+H(x,\;y_{2})$$

subject to *d*_min_ ≤ *y*_2_ + *j*_2_ – *y*_1_ – *j*_1_ ≤ *d*_max_, *k* = *d_r_*, and 2 ≤ *x* ≤ *N*; where α and β are weighing factors of the line smoothness. All tuples (*y*_1_, *y*_2_) are tested if they fit the constraints. Actually, both Δω*_x_* and (|*j*_1_| + |*j*_2_|) can form a (2*k* + 1) × (2*k* + 1) matrix. Take k = 1 as an example as follows:

$${\Delta \omega }_{x}=\left[\begin{array}{ccc} 0 & 1 & 2\\ 1 & 0 & 1\\ 2 & 1 & 0 \end{array}\right],\;\;\text{and}(|j_{1}|+|j_{2}|)=\left[\begin{array}{ccc} 2 & 1 & 2\\ 1 & 0 & 1\\ 2 & 1 & 2 \end{array}\right]$$

Therefore, ${\alpha \Delta \omega }_{x}+\beta (|j_{1}|+|j_{2}|)=\left\lfloor \begin{array}{ccc} 2\beta & \alpha +\beta & 2(\alpha +\beta )\\ \alpha +\beta & 0 & \alpha +\beta \\ 2(\alpha +\beta ) & \alpha +\beta & 2\beta \end{array}\right\rfloor$, where the row and column index pair is (*j*_1_, *j*_2_). Since the minimization problem depends on (*j*_1_, *j*_2_) only, the process can be accelerated via finding the minimum in the following matrix:

$$\begin{array}{l} J(x-1,\;y_{1}+j_{1},\;y_{2}+j_{2})+{\alpha \Delta \omega }_{x}+\beta (|j_{1}|+|j_{2}|)=\\ \left[\begin{array}{ccc} J(x-1,\;y_{1}-1,\;y_{2}-1) & J(x-1,\;y_{1}-1,\;y_{2}) & J(x-1,\;y_{1}-1,\;y_{2}+1)\\ J(x-1,\;y_{1},\;y_{2}-1) & J(x-1,\;y_{1},\;y_{2}) & J(x-1,\;y_{1},\;y_{2}+1)\\ J(x-1,\;y_{1}+1,\;y_{2}-1) & J(x-1,\;y_{1}+1,\;y_{2}) & J(x-1,\;y_{1}+1,\;y_{2}+1) \end{array}\right]+\left[\begin{array}{ccc} 2\beta & \alpha +\beta & 2(\alpha +\beta )\\ \alpha +\beta & 0 & \alpha +\beta \\ 2(\alpha +\beta ) & \alpha +\beta & 2\beta \end{array}\right] \end{array}$$

This can be expanded to a (2*k* + 1) × (2*k* + 1) matrix if k>1. The initial values of *J*(1, *y*_1_, *y*_2_) is defined by *J*(1, *y*_1_, *y*_2_) = *H*(1, *y*_1_) + *H*(1, *y*_2_) and the optimal tuple ( $j_{1}^{*}$, $j_{2}^{*}$) can be determined by searching the minimum in the (2*k* + 1) × (2*k* + 1) matrix: ( $j_{1}^{*}$, $j_{2}^{*}$) = arg min_(_*_j_*_1,_ *_j_*_2)_ *J*(*x*, *y*_1_ + *j*_1_, *y*_2_ + *j*_2_). The indices pointing backwards are stored in coordinate matrices C1 and C2 as follows:

(6) $$\begin{array}{l} C1(x,\;y_{1},\;y_{2})=y_{1}+j_{1}^{*}\\ C2(x,y_{1},y_{2})=y_{2}+j_{2}^{*} \end{array}$$

After the DDP is processed, the cost values are computed and saved in the cost map *J*(*x*, *y*_1_, *y*_2_) ∈ *R*^3^. The minimum value in the last matrix, *i.e.*, *J*(*N*, *y*_1_, *y*_2_) is searched, which indicates the last points of the dual lines. Having a backwards tracking from N to 1 in the coordinate matrices C1 and C2, the complete coordinates of the dual lines can be obtained.

### 3.4. Artery Movement Tracking (AMT)

The artery has extension and possible movement during the heart cycle. The movement is mostly in the y-direction, *i.e.*, orthogonal to the artery flow. This is because the pressure from the upper skin is less than the pressure from the tissues below. Therefore, the ROI is changing in each image of a sequence. A simple automatic tracking scheme is embedded in order to track a correct ROI for the following DDP process. Let *T*_0_(*x* + *x*_0_, *y* + *y*_0_) ∈ *R*^2^ be the rectangular ROI selected by the user in the first image of a given sequence, where 1 ≤ *x* ≤ Δ*x*, 1 ≤ *y* ≤ Δ*y*, Δ*y* × Δ*x* is the size of the ROI, and (*x*_0_, *y*_0_) is a reference point (*i.e.*, the upper-left corner of the ROI) on the first image. The normalized correlation coefficient (NCC) is applied to find out the shift in y-direction on each subsequent image:

(7) $$y_{i,s}^{*}=\underset{-\Delta s\leq s\leq \Delta s}{\text{max}}\frac{1}{\sigma_{T_{0}}\sigma_{T_{i,s}}}\sum_{y=1}^{\Delta y}\sum_{x=1}^{\Delta x}\left(T_{0}(x+x_{0},\;y+y_{0})-{\hat{T}}_{0})(T_{i}(x+x_{0},\;y+y_{i-1}+s)-{\hat{T}}_{i,s}\right)$$

where *i* denotes image number; (*x_i_*_–1_, *y_i_*_–1_) is the reference point in image (*I* − 1); *T̂*_0_ and *T̂_i,s_* are the mean gray values of the regions *T*_0_ and *T_i_*(*s*), respectively; σ_*T*_0__ and σ_*T_i,s_*_ are the standard deviations of the regions *T*_0_ and *T_i_*(*s*), respectively; Δs is a small integer covering a possible range of artery movement in the y-direction. The optimal value is the maximal NCC. Thus, $y_{i}=y_{i-1}+y_{i,s}^{*}$.

### 3.5. Procedure

We summarized the whole process in Section 3 to be a procedure as follows:

Step 1: The user selects a directory in the hard disk where images of an image sequence are unpacked and stored.
Step 2: The image number (L) in the directory is counted. The first image is automatically loaded and shown on the monitor.
Step 3: The user selects a rectangular area where the artery should be analyzed including the near and the far wall of the CA.
Step 4: The selected rectangular area is equally divided into two parts: the upper and lower parts. Record the coordinates. To analyze near wall, go to Step 5. To analyze far wall, go to Step 6.
Step 5:
  (a) Let *I* denote the upper part of the rectangular area, *I* ∈ *R^M^*^×^*^N^*. Normalize *I* so that 0 ≤ *I* ≤ 1. *F* = *I* *f_1_, where *f*_1_ = [−1,−1,−1,−1,0,1,1,1,1] is a column vector. Normalize F so that 0 ≤ *F* ≤ 1.
  (b) Set *d*_min_ = 0, *d*_max_ = 1, *k* =1, apply DDP to find out the outer wall coordinate *y_outer_*.
  (c) Re-calculate F using (1), where *f_x_* = −*f_IMT_*_(_*_far_*_)_. Normalize F so that 0 ≤ *F* ≤ 1. Set H to **1**, *H* ∈ *R^M^*^×^*^N^*. Calculate H using (3).
  (d) Combine F and H together: H=1-F+cH, where c is a weighting factor.
  (e) Set *d*_min_ = 4, *d*_max_ = 0.9*M*, *k* =1, apply DDP to find out the intima and adventitia coordinates of the near wall. Record the coordinates.
  (f) Set i = i + 1. If i > L, end the procedure, else input next image and use AMT in Section III to track the correct position of the near wall. Output *I* and go to Step 5 (a).
Step 6:
  (a) Let *I* denote the lower part of the rectangular area, *I* ∈ *R^M^*^×^*^N^*. Normalize *I* so that 0 ≤ *I* ≤ 1. Calculate F using (1). Normalize F so that 0 ≤ *F* ≤ 1. Set H to **1**, *H* ∈ *R^M^*^×^*^N^*.
  (b) Set *d*_min_ = 4, *d*_max_ = 0.9 *M*, *k* = 1, apply DDP to find out the intima and adventitia coordinates of the near wall. Record the coordinates.
  (c) Set i = i + 1. If i > L, go to Step 7, else input next image and use AMT in Section III to track the correct position of the near wall. Output *I* and go to Step 6 (a).
Step 7: Smooth the output intima and adventitia lines using averaging technique and show it on the images.

### 3.6. Accuracy

An accuracy study is an assessment of the reliability of a system. In order to compare results made by the proposed automated system, we developed software for manual drawings. The manual drawings were performed by well-trained radiology technicians and then confirmed and modified by a medical physician. Four layers were drawn, *i.e.*, intima and adventitia of both near and far walls. The linear interpolation is applied to fill out points between two points in manual drawings.

Assume a layer goes from left to right (*x*_1_ to *x_N_*) and the *i*-th corresponding y-coordinate of the manual drawing and automated result are *y_i_* and *ŷ_i_*, respectively. The averaged error of one layer is computed by $e=\frac{1}{N}\sum_{i=1}^{N}\left|y_{i}-{\hat{y}}_{i}\right|$. Since each image sequence has many static images, the total averaged error of a sequence is $E=\frac{1}{M}\sum_{1}^{M}e_{i}$, where there are M static images in the sequence.

## 4. Results

The B-mode sonographic image videos were captured by an experienced physician of the Institute of Exercise and Health Sciences Vascular Lab at Basel University. Each video has more than 78 static images after decoding. The image resolution is 652 × 800 and the pixel size is about 0.106 mm. We took three test videos and show some of them in the paper.

The IMT detection has duality, *i.e.*, near and far wall IMT detections. Traditionally, the far wall is taken for IMT measurement because the near wall intima is insufficiently visible due to the noise impact in many cases. Our method has better robustness against noise impact on the near wall and it is able to detect IMT on both near and far walls with and without plaques. Moreover, the measurement of LD is the distance between both intima of near and far walls.

**Figure 6.**
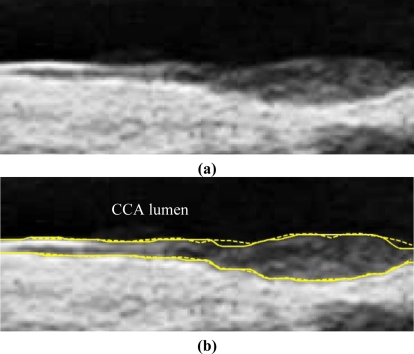
Far wall IMT detection. **(a)** Raw sub-image. **(b)** Solid line: Automatic detection result. Dash line: Manual drawing. (Sequence S1).

**Figure 7.**
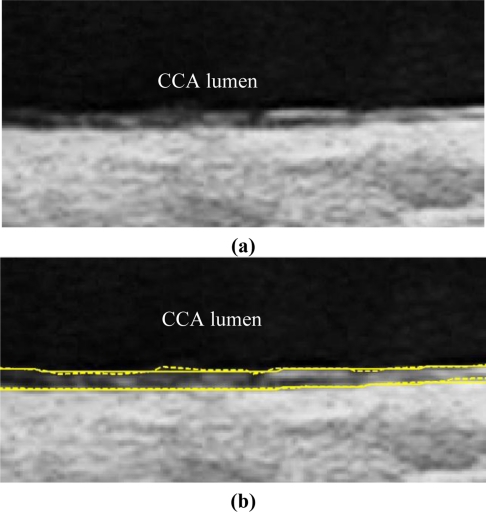
Far wall IMT detection. **(a)** Raw sub-image. **(b)** Solid line: Automatic detection result. Dash line: Manual drawing. (Sequence S2).

**Figure 8.**
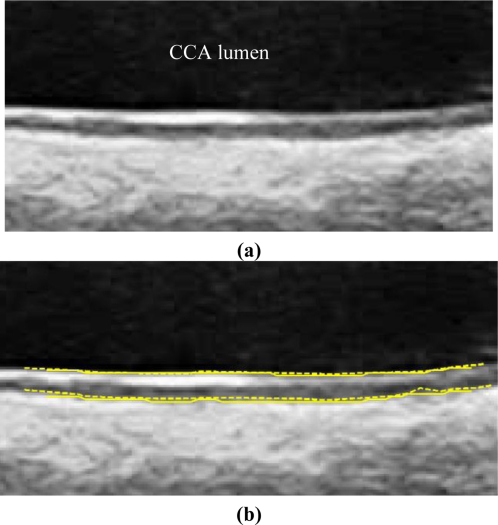
Far wall IMT detection. **(a)** Raw sub-image. **(b)** Solid line: Automatic detection result. Dash line: Manual drawing. (Sequence S3).

**Figure 9.**
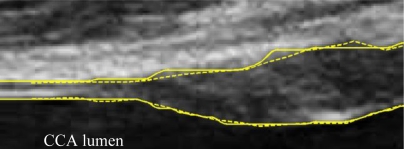
There is a plaque on the right-hand side. The left-hand side has a normal thickness. Solid line: automatic detection result. Dash line: Manual drawing. (Sequence S1).

**Figure 10.**
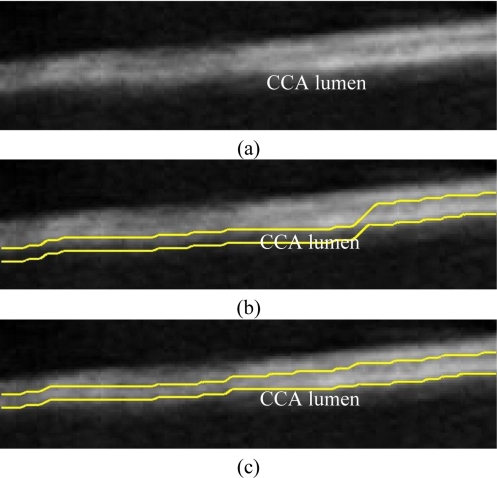
**(a)** Sub-image. **(b)** Result made by [7]. **(c)** Result of the proposed method. (Sequence S2).

**Figure 11.**
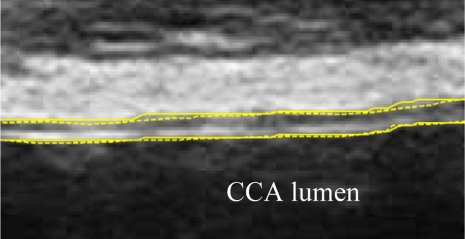
Result superimposed on the raw sub-image. Solid line: automatic detection result. Dash line: Manual drawing. (Sequence S3).

We have tested three image sequences: S1, S2, and S3, they include plaques, thin, and thick IMT, respectively. All sequences have at least three heart cycles. Figures 6 to 8 show the test results of IMT detection on the far wall, whereas Figures 9 to 11 show the test results of IMT detection on the near wall of the three sequences.

In Figure 6 there is a plaque on the right-hand side which is successfully detected. A total of 78 images are in sequence S1. Another example shows the ability to detect a thin IMT as shown in Figure 7. A total of 111 images are in sequence S2. Figure 8 shows the thick IMT test result. A total of 86 images are in sequence S3.

Figure 9 shows that our method has the ability to detect the plaque on the near wall. Figure 10(b) shows the problem using the method in [7] to detect the dual lines when the near wall is not clear. However, the proposed method can have a good result, as shown in Figure 10(c). As well, it produces good results in thicker IMT detection (Figure 11).

**Figure 12.**
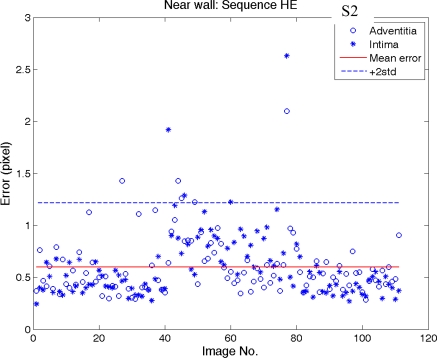
“*” and “o” denote the near wall error distribution of intima and adventitia along image number, respectively. The total averaged error of the near wall is 0.6-pixel in sequence S2.

**Figure 13.**
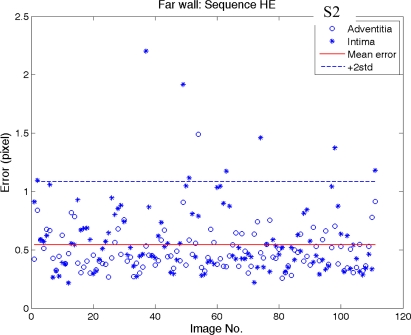
“*” and “o” denote the far wall error distribution of intima and adventitia along image number, respectively. The total averaged error of the far wall is 0.54-pixel in sequence S2.

Figures 12 and 13 show the error distributions of intima and adventitia along image frame number of near and far wall (sequence S2), respectively. The error distributions of the other two sequences are similar and are not shown here.

Figure 12 shows an increase of error during the systolic phase of the heart cycle in the near wall. During this phase the wall movements usually is strongest and results in a blurring of the arterial wall. In the sequence S2 the systolic cycle begins at image 4, 41, 77, and 111. In the middle two heart cycles (begins at image 41 and 77) the artery movement is significant.

It can be noted that there are two “hills” during this period. It reveals that intima has a lower intensity than the adventitia, which results in a longer blurring (image 40–80) than in the adventitia (image 40–60). However, this error can be treated as low because its averaged error during these two “hills” is around 0.8 pixels. Compare this to the total average error (0.6 pixel for near wall and 0.55 pixel for far wall in Table 1) the difference (between 0.8 pixel and 0.55 pixel) is only 0.25 pixel (0.0265 mm). Absolutely it is in the range of 0.048 mm, thus within 5% of the IMT.

We summarize all errors of the automated results comparing to the expert’s manual tracing in Table 1. The average error on near wall detection (0.63 pixel or 0.067 mm) is larger than the far wall detection (0.55 pixel or 0.058 mm). This is because the near wall is in general vaguer than the far wall, especially in the systolic cycle. The intima detection is in general easier than the adventitia detection if there is a plaque. In thin or thick IMT cases the difference is not significant.

In order to test our system, we analyzed more patients’ image sequences (S4–S7) and calculated the unsigned (absolute error) and signed error. In this experiment we detect intima on both near and far walls. This is because we want to analyze the artery’s lumen diameter change with respect to time during the heart cycle. The unsigned (Equation 8a) and signed (Equation 8b) error in *j*-th frame, similar to the equation in section 3.6, are calculated using the equations:

(8a) $$e_{j}=\frac{1}{N}\sum_{i=1}^{N}\left|y_{\text{Automatic}}(i)-y_{\text{Manual}}(i)\right|$$

(8b) $${\hat{e}}_{j}=\frac{1}{N}\sum_{i=1}^{N}\left(y_{Automatic}(i)-y_{Manual}(i)\right)$$

where *y_Automatic_(i)* and *y_Manual_(i)* denote the i-th y-coordinate of automatic result and manual drawing. However, N is limited to 10 points in each image because the manual drawing is a tremendous human work. The averaged error in a sequence is calculated by:

$$E_{\text{unsigned}}=\frac{1}{M}\sum_{j=1}^{M}e_{j}\;\;\;\text{and}\;\;E_{\text{signed}}=\frac{1}{M}\sum_{j=1}^{M}{\hat{e}}_{j}.\;\;\;(M=21)$$

The results are shown in Table 2.

From Table 2 it is noted that the signed error at the near wall is larger than that for the far wall. The far wall has almost no bias between the automated results and the manual drawings. This confirms that the detection on near walls is in general more difficult than the detection on far walls.

**Figure 14.**
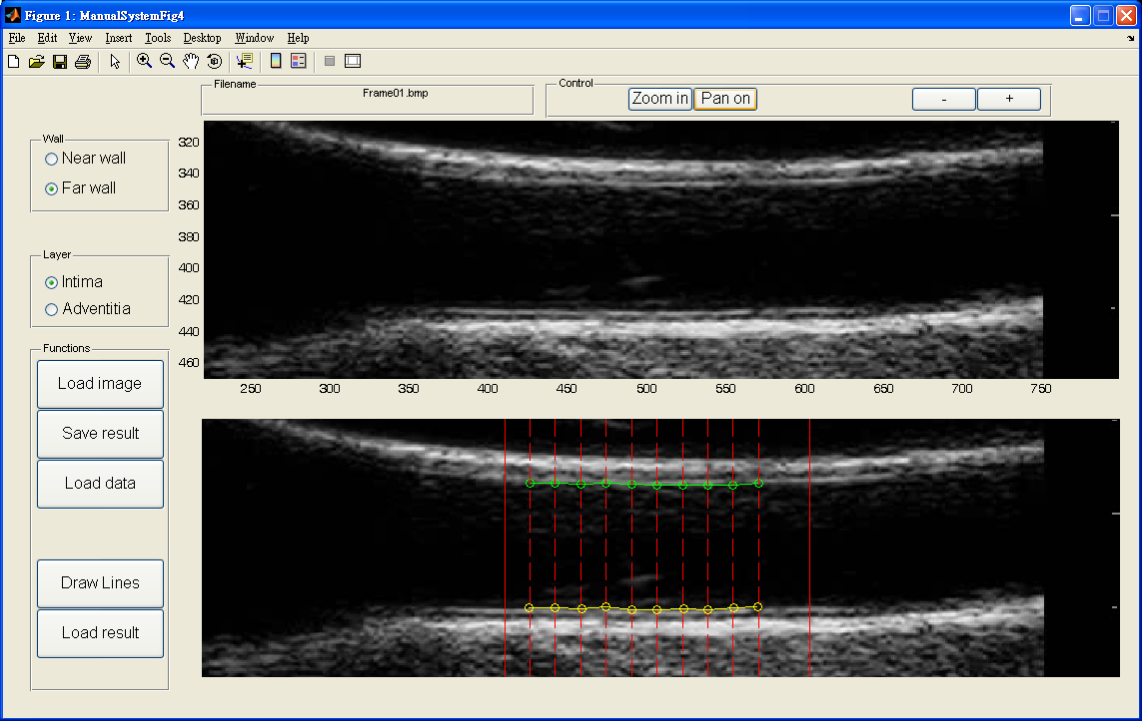
The GUI for manual drawing. The dash lines denote where the expert should give points.

Figure 14 depicts our manual drawing GUI. The solid lines denote the boundary of the automated analysis. The dashed lines denote where the expert should give their judgment on the lumen-intima interface. Manual drawing involves a tremendous amount of human work. Although we are not able to provide many manual drawings for comparison, however, the automated results were controlled visually by experts and all results are similar to those shown in Tables 1 and 2. The system is built on the Matlab platform. Some kernel functions are written in C language in order to reduce the computation time.

## 5. Discussion

According to our experience, the most difficult part is to detect the intima as well as the adventitia of the near wall. This is due to the fact that the near wall has a greater moving speed than that on the far wall during the systolic and diastolic cycle.

The dynamic analysis of the IMT and lumen of the artery faces problems which do not occur with static images. The static image is a single shot providing a clear view of the intima-lumen and adventitia-media interfaces. During the heart cycle the near wall usually moves more than 1 mm and might thus be associated with the blurring of the IM layers. This usually affects the intima-lumen interface more than the adventitia-medial interface due to the lower echogeneity of the intima. In CCA near walls with a thinner IMT this problem might also happen in the adventitia, as shown in Figure 10. However, the tracing error is relatively low compared with the overall movement of the artery. Notably, the accuracy of the detection on intimal layer does not show significant difference between the cases which have or have not plaque. Therefore, the existence of plaque does not affect the accuracy in calculation of the lumen diameter.

The artery adventitia is difficult to recognize if a plaque exists. For instance, the formation of a vulnerable plaque might be due to a severe microbial invasion. The microbial products and autoantibodies stick to the lipoproteins, which aggregate and obstruct the capillary lumen, leading to local ischemia and inflammation [19]. This may result in a blurred boundary between media and adventitia. This might be one of the reasons that the accuracy of automated identification on adventitia in the sequence S1 is worse than the other two sequences (Table 1). Precise plaque detection is a prerequisite for the calculation of total plaque area. For automatic detection, noises within a plaque do seriously disturb the tracing process.

The main contributions of this study are: (1) we propose a scheme which is able to detect the near and far wall intima and adventitia with and without plaque; (2) we solve the ambiguity problem caused by artery movement. These are the two major problems in the artery wall dynamic analysis in the B-mode RF sensors. Dynamic analysis of the artery lumen area might be a direct way to measure the local compliance and distensibility of the common carotid artery. Due to the heterogeneity of the arterial wall, the measurement at a single line—as it is in traditional M-mode imaging—may not be representative for the elastic properties of the arterial across a segment. The measurement of IMT as well as the LD in B-Mode image series might 1. Be more representative and 2. Offer the opportunity to show differences within a certain segment which stand for different atherosclerotic processes.

This may not be true for the plaque. However, to show changes of plaque thickness over time might be an indicator for vulnerability of the plaque, which is of significant clinical importance with respect to future cardio-vascular events.

## 6. Conclusions

A new method of analyzing IMT on both near and far wall of CCA in dynamic B-mode sonographic image sequences is proposed. This method is able to detect thin or thick IMT, with or without plaques. We have tested three image sequences and all results are compared to the manual tracing made by two experts. We have compared this method with the previous study and the results show that this method has better performance. The average error is of a sub-pixel level, *i.e.*, 0.55 pixel (0.0583 mm) for the far wall and 0.63 pixel (0.0668 mm) for the near wall detection.

## Figures and Tables

**Table 1.** Averaged errors of proposed method comparing to manual drawings.

**Sequence name**	**Near wall (Unit: pixel)**			**Far wall (Unit: pixel)**			**Quantity of images**
	**Intima**	**Adventitia**	**Average**	**Intima**	**Adventitia**	**Average**	
S1 S2 S3 Average	0.58 0.60 0.50 0.56	0.97 0.60 0.54 0.69	0.78 0.60 0.52 0.63	0.53 0.60 0.47 0.53	0.72 0.49 0.47 0.56	0.63 0.55 0.47 0.55	78 111 86

1 pixel = 0.106 mm

**Table 2.** Averaged unsigned and signed error. The comparison between automated result and expert’s manual drawing. (1 pixel = 0.106 mm).

**Sequence name**	**Averaged unsigned error in pixel: *E_unsigned_***		**Averaged signed error in pixel: *E_signed_***	
	**Near wall**	**Far wall**	**Near wall**	**Far wall**
S4 S5 S6 S7	0.67 0.83 0.89 0.54	0.46 0.42 0.40 0.39	0.23 0.21 0.60 −0.22	0.02 0.04 0.05 −0.02
